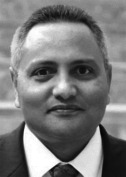# Oxygen‐Deficient Cobalt‐Based Oxides for Electrocatalytic Water Splitting

**DOI:** 10.1002/cssc.202002002

**Published:** 2020-12-04

**Authors:** Ahmed Badreldin, Aya E. Abusrafa, Ahmed Abdel‐Wahab

**Affiliations:** ^1^ Chemical Engineering Program Texas A&M University at Qatar P.O. Box 23874 Doha Qatar

**Keywords:** cobalt oxides, oxygen-deficient electrocatalysts, oxygen evolution reaction, oxygen vacancies, water electrolysis

## Abstract

An apparent increased interest has been recently devoted towards the previously untrodden path for anionic point defect engineering of electrocatalytic surfaces. The role of vacancy engineering in improving photo‐ and electrocatalytic activities of transition metal oxides (TMOs) has been widely reported. In particular, oxygen vacancy modulation on electrocatalysts of cobalt‐based TMOs has seen a fresh spike of research work due to the substantial improvements they have shown towards oxygen evolution reaction (OER) and hydrogen evolution reaction (HER). Oxygen vacancy engineering is an effective scheme to quintessentially tune the electronic structure and charge transport, generate secondary active surface phases, and modify the surface adsorption/desorption behavior of reaction intermediates during water splitting. Based on contemporary efforts for inducing oxygen vacancies in a variety of cobalt oxide types, this work addresses facile and environmentally benign synthesis strategies, characterization techniques, and detailed insight into the intrinsic mechanistic modulation of electrocatalysts. It is our foresight that appropriate utilization of the principles discussed herein will aid researchers in rationally designing novel materials that can outperform noble metal‐based electrocatalysts. Ultimately, future electrocatalysis implementation for selective seawater splitting is believed to depend on regulating the surface chemistry of active and stable TMOs.

## Graphical abstract

**Lattice oxygen out, overpotential down**: A clear reduction in electrocatalytic oxygen evolution reaction overpotential is typically witnessed upon fine‐tuning the degree of oxygen vacancies within a transition metal oxide lattice. Cobalt‐based metal oxides (Co−MO) are amongst the most promising electrocatalysts for water electrolysis. This Review offers a template for capitalizing on the realm of oxygen deficiency engineering atop Co−MO to reinforce their electrocatalytic performance.

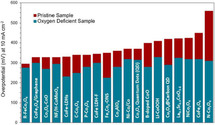

## 1 Introduction

The ever‐growing energy demand and the depletion of traditional fossil fuels have spurred continuous research efforts to deploy clean and renewable alternative energy resources such as solar energy and wind power.^[1]^ Also, there is a pressing need to tackle environmental problems associated with carbon dioxide (CO_2_) emissions, which is the major cause of global warming. However, the intermittent shortcomings of the aforementioned renewable energy resources have limited their widespread deployment. An especially appealing approach to overcome this issue is to convert and store solar and wind‐derived energy in the form of chemical bonds such as hydrogen.^[2]^

Hydrogen (H_2_) is an ideal energy carrier and has been widely conceded as a clean and sustainable carbon‐free energy source to replace non‐renewable carbon‐emitting fossil fuels owing to its high specific energy and environmental compatibility.[^1a^, ^3^] As such, electrocatalytic water splitting using electricity produced from renewable energy sources is a promising approach to produce high‐purity hydrogen with zero carbon footprint.^[4]^ Generally, the process occurs in either alkaline or acidic electrolyte via two half‐reactions, the hydrogen evolution reaction (HER) at the cathode and the oxygen evolution reaction (OER) at the anode.^[5]^ Theoretically, to drive electrochemical water splitting, a thermodynamic cell potential of 1.23 V [vs. reversible hydrogen electrode (RHE)] is required under standard conditions, corresponding to an energy input of 237.1 kJ mol^−1^.^[6]^ However, in practice, to drive the water‐splitting process at an appreciable rate, a considerable excess potential denoted as overpotential must be applied to overcome the activation energy barriers due to the sluggish reaction kinetics, especially for the OER.^[1c]^ This has significantly hampered the development and widespread implementation of electrocatalytic water‐splitting at a large scale. Currently, the contribution of electrochemical water splitting processes to the overall global hydrogen production is only 4 %.[^6^, ^7^] Therefore, further improvements are required to overcome the bottlenecks associated with water‐splitting technologies to satisfy the global demand for clean and sustainable hydrogen production.^[8]^ This is realized through the rational design of highly stable and active electrocatalysts to facilitate the reaction kinetics and to minimize the required energy input.[^7^, ^8^, ^9^]

To date, various electrocatalytic systems have been reported to overcome the limitations associated with the overall water‐splitting reactions, especially for the OER. Currently, the benchmark electrocatalysts for water splitting are noble metals such as platinum (Pt) for HER,^[10]^ and ruthenium dioxide (RuO_2_)/iridium dioxide (IrO_2_) for OER.^[11]^ However, the high cost and scarcity of noble metal‐based electrocatalysts limit their potential use in commercial‐scale hydrogen production. Thus, considerable research efforts have been devoted to exploit efficient, active, and cost‐effective electrocatalysts that are composed of earth‐abundant materials to replace noble metal‐based electrocatalysts. Transition metal oxide‐based materials (TMOs) (e. g., rocksalt oxides, spinel oxides, rutile oxides, perovskites) have attracted tremendous attention as suitable electrocatalysts especially towards OER due to their abundancy, low cost, corrosion resistance, and ease of synthesis.^[12]^ Furthermore, TMOs exhibits multivalence oxidation states, which act as active sites for the electrocatalytic water splitting.^[12b]^

Among TMO materials, cobalt‐based electrocatalysts have been widely promoted as potential non‐precious metal OER and HER electrocatalysts for water splitting.^[13]^ Cobalt (Co) is the 32nd most abundant element in the Earth's crust, and it is a fairly inexpensive material. However, the relatively poor conductivity and low electrocatalytic activity of Co‐based TMOs compared with benchmark noble metal‐based electrocatalysts limit their practical applications.^[12b]^ Thus, despite their promise, TMOs still require further improvement to outperform current benchmark OER and HER electrocatalysts. Design of materials that promote water dissociation and provide moderate adsorption‐desorption behavior of HER and OER intermediates, all of which depend on the electronic structure, can effectively enhance the overall water splitting performance.^[1a]^ Particular focus is typically given to cobalt‐based metal oxides due to the abundance of feasible structures that can be fabricated, low cost of base materials, high relative stabilities in alkaline environment especially at high current densities, variable chemical states possible for cobalt, which allow an abundance for surface energetics modulation, and intrinsically high activities at near‐neutral pH.^[14]^ The latter is of paramount importance for electrolysis of seawater as an industrially appropriate means of hydrogen production from an abundant feed water source. The feasibility of operating the electrolyzers at high current densities and near‐neutral pH conditions substantially brings down operational costs and facilitates the utilization of seawater electrolysis as a competitive method for effective and sustainable hydrogen generation, especially in regions that have limited freshwater availability.

Engineering surface defects in general and oxygen‐vacancies in particular has been increasingly explored as an effective strategy to modulate the electronic structure of TMOs toward improving electrocatalytic water splitting performance.[^12c^, ^15^] Experimental and theoretical analysis proved that oxygen defects in TMOs can favorably promote fast charge transfer, reduce kinetic energy barriers, and provide moderate adsorption‐desorption of H_2_O and intermediate reaction species (H^+^ for HER; M−OH, M−OOH, M−O for OER), leading to substantial HER and OER activity improvement.[^7^, ^16^] Several recent reports demonstrated that oxygen vacancies at the atomic level increase the density of active sites through the formation of low coordination metal cations, which act as electrocatalytic active sites.^[17]^ The formation of low‐valence metal cations promotes the adsorption of OH^−^ and H_2_O (i. e., lowers the energy barrier for H_2_O dissociation) and facilitates its conversion to other active oxyhydroxide intermediates;^[6]^ this ultimately enhances both HER and OER performances. Induction of oxygen vacancies can effectively ameliorate intrinsic electrochemical challenges of a pristine sample and consequently substantially improve electrocatalytic activity, as was the case with Sr_2_VFeAsO_3‐*δ*_, which showed 80 times higher specific activity at 1.7 V vs. RHE at *δ*=0.5.^[18]^

Moreover, upon introducing oxygen vacancies the bandgap narrows, and new gap states form within the bandgap near the Fermi level. A narrow gap between the metal 3d and oxygen 2p band centers provides covalent bonding between the reaction intermediates and the catalyst.^[17]^ Furthermore, upon removing an oxygen atom from the TMO lattice, the two electrons that formerly occupied the oxygen 2p orbitals tend to delocalize around the metal cation adjacent to the oxygen vacancy. The electrons in the delocalized electronic structure can be easily excited into the conduction band,^[19]^ which leads to faster charge separation and transfer (enhanced conductivity). All the aforementioned effects minimize the overpotential and facilitate electrochemical water splitting.[^7^, ^16a^, ^16b^, ^20^]

Oxygen vacancies were reported to alter the electron configuration of the transition metal cations to the high spin state through e_g_ orbital filling (near‐unity filling is optimum), which can easily form bonding with OH^−^. The e_g_ orbital has a stronger overlap with oxygenated adsorbates than does the π‐bonding t_2g_ orbital.^[21]^ Thus, the overlapping of the e_g_ orbital on the transition metal cation with the O‐p σ‐orbital on OH^−^ promotes the formation of intermediate M−OH and enhances ion transfer between the metal cation and the adsorbed reaction intermediates. Moreover, high spin electron configuration promotes the electrophilicity of the adsorbed M−O and thus accelerates the reaction between M−O and OH^−^ to form M−OOH intermediate, which is typically a rate‐determining step in OER.^[22]^ These effects contribute to lowering the kinetic energy barriers of the multi elementary steps in OER and accelerates the reaction kinetics.^[23]^

Significant efforts towards the preparation of oxygen‐deficient Co‐based TMOs have been extensively reported in the literature. Different oxygen‐deficient TMOs reported in the literature for OER electrocatalysts and for HER and bifunctional electrocatalysts are summarized in Tables 1 and 2, respectively. Herein we provide a thorough overview of recent advancements towards exploiting oxygen‐deficient TMOs for efficient electrochemical water splitting. Synthesis approaches reported to induce vacancies in Co‐based TMOs and their effects on surface morphology, crystallinity, and electrocatalytic OER and HER activity are summarized. Moreover, advanced identification techniques that have been widely employed to detect and characterize oxygen vacancies are presented. Also, experimental and theoretical explanations on the influence of oxygen vacancies on active site density, electronic structure, and kinetic energy barriers, as well as their role in minimizing HER and OER overpotentials are discussed. Finally, key challenges and prospects for new directions towards developing potential Co‐based TMOs electrocatalytic systems to further enhance OER and HER activity are proposed.

**Table 1:** Summary of major oxygen‐deficient Co‐based TMOs electrocatalysis for OER.

Synthesis method	Material	Method	Overpotential (b.t.) [mV]	Overpotential (a.t.) [mV]	Tafel slope (b.t.) [mV dec^−1^]	Tafel slope (a.t.) [mV dec^−1^]	KOH [m]	Ref.
thermal treatment	B‐CoO nanowires	CoO precursor was obtained by hydrothermal reaction of [Co (Ac)_2_ ⋅ 4H_2_O], (NH_4_HB_4_O_7_ ⋅ 3H_2_O) and water at 200 °C for 10 h, was annealed in Ar at 500 °C for 2 h	400 at 10 mA cm^−2^	280 at 10 mA cm^−2^	71	86	1	[24]
thermal treatment	Ni−Co/Co hydroxides	pristine Ni−Co/Co hydroxides were annealed in air at 300 °C	353 at 10 mA cm^−2^	320 at 10 mA cm^−2^	57	30	1	[25]
thermal treatment	PrBaCo_2_O_5.75_ PrBaCo_2_O_5.5_ perovskite	polycrystalline powders of PrBaCo_2_O_6‐*δ*_ were annealed in N_2_ atmosphere at 250 and 600 °C for 30 min	–	360 (PrBaCo_2_O_5.75_) 420 (PrBaCo_2_O_5.5_) at 10 mA cm^−2^	–	70 (PrBaCo_2_O_5.75_) 80 (PrBaCo_2_O_5.5_)	1	[26]
hydrogen treatment	Fe_2_O_3_/CoO_*x*_	pristine Fe_2_O_3_/CoO_*x*_ composite was annealed in 10 %H_2_/Ar at 150–300 °C for 30 min	–	316 at 10 mA cm^−2^	–	56	1	[27]
hydrogen treatment	Co_3_O_4_ quantum dots	pristine Co_3_O_4_ quantum dots were reduced 5 %H_2_/95 %Ar at 170 and 200 °C, 1 h	370 at 10 mA cm^−2^	315 (Co_3_O_4_‐200) 348 (Co_3_O_4_‐170) at 10 mA cm^−2^	72	49 (Co_3_O_4_‐200) 66 (Co_3_O_4_‐170)	1	[28]
hydrogen treatment	Ca_2_Mn_2_O_5_ perovskite	pristine CaMnO_3_ was annealed in 5 %H_2_ in Ar at 350 °C for 3 h	500 at 0.5 mA cm^−2^	380 at 1 mA cm^−2^	197	149	0.1	[23]
wet‐chemical reduction	Co_3_O_4_ mesoporous wires	pristine mesoporous Co_3_O_4_ NWs were reduced with NaBH_4_ solution at RT for 1 h	370 at 1.8 mA cm^−2^	370 at 13.1 mA cm^−2^	82	72	1	[16b]
wet‐chemical reduction	Fe_*x*_Co_*y*_‐ONS nanosheets	NaBH_4_ aqueous solution was added to a solution containing [Co(NO_3_)_2_, Fe(NO_3_)_3_, cetyltrimethylammonium bromide] at RT for 5 min	40 at 10 mA cm^−2^	308 at 10 mA cm^−2^	71.7	36.8	0.1	[29]
wet‐chemical reduction	Co_2_AlO_4_ nanosheets	pristine Co_2_AlO_4_ nanosheets were treated in a homogeneous solution of NaOH and ethylene glycol at 140 °C for 12 h	360 at 10 mA cm^−2^	280 at 10 mA cm^−2^	79.98	70.98	1	[30]
wet‐chemical reduction	CoFe_2_O_4_/graphene composite	pristine CoFe_2_O_4_/graphene was reduced with NaBH_4_ solution for 2 h	330 at 10 mA cm^−2^	300 at 10 mA cm^−2^	67	68	1	[31]
plasma treatment	Co_3_O_4_‐CoO nanosheets	Co_3_O_4_ nanosheets were treated by Ar–H_2_ plasma	330 at 10 mA cm^−2^	270 at 10 mA cm^−2^	49.3	49	1	[32]
plasma treatment	N‐doped Co_3_O_4_ nanosheets	pristine Co_3_O_4_ nanosheets were treated with N_2_ plasma at 300 W, 40 Pa for 60–80 s	560 at 10 mA cm^−2^	310 at 10 mA cm^−2^	234	59	0.1	[33]
plasma treatment	CoFe LDHs nanosheets	pristine CoFe LDHs were treated with H_2_O plasma with a dielectric barrier discharge (DBD) plasma reactor for 2–15 min	332 at 10 mA cm^−2^	290 at 10 mA cm^−2^	52	36	1	[34]
hydro‐thermal treatment	P‐doped Co_3_O_4_ nanosheet	Co_3_O_4_ and NaH_2_PO_2_⋅H_2_O with different weight ratios (2, 15, 30) were annealed in Ar atmosphere at low static pressure and at 250 °C for 1 h	395 at 20 mA cm^−2^	338 at 20 mA cm^−2^	62	52	1	[35]
laser irradiation	L–CoOOH	Co target was immersed in KOH solution was irradiated by nanosecond laser for 20 min at RT	410 at 10 mA cm^−2^	330 at 20 mA cm^−2^	75.4	63.2	1	[36]
laser irradiation	R‐FeCo_2_O_4_ nanoparticles	a water suspension of M‐FeCo_2_O_4_ microspheres was irradiated by laser for 30 min at RT	297 at 10 mA cm^−2^	276 at 10 mA cm^−2^	72.1	70.8	0.1	[37]
plasma treatment	La_0.7_Sr_0.3_CoO_3‐*δ*_	pristine La_0.7_Sr_0.3_CoO_3‐*δ*_ was treated in Ar plasma for 10 min at 300 W and 70 Pa	433 at 10 mA cm^−2^	326 at 10 mA cm^−2^	92.1	61.6	1	[38]
thermal treatment	B‐doped CoO	a mixture of Co (Ac)_2_ and NH_4_HB_4_O_7_ (hydrothermally treated at 200 °C for 10 h) was annealed at 500 °C for 2 h in Ar	400 at 10 mA cm^−2^	280 at 10 mA cm^−2^	86	71	1	[24]
wet‐chemical reduction	CoFe_2_O_4_ nanosheets	pristine CoFe_2_O_4_ hollow nanospheres were reduced with NaBH_4_ solution for 1 h at RT	450 at 10 mA cm^−2^	320 at 10 mA cm^−2^	94	48	1	[39]
hydrogen treatment	reduced CaMn_0.5_Nb_0.25_O_3‐*δ*_	pristine CaMn_0.5_Nb_0.25_O_3‐*δ*_ was annealed under 30 % H_2_/N_2_ at 350 °C for 2 h	300 at 0.5 mA cm^−2^	280 at 0.5 mA cm^−2^	140	98	0.1	[40]
ball milling	(Co_0.2_Mn_0.2_Ni_0.2_Fe_0.2_Zn_0.2_) Fe_2_O_4_	CoO, MnO, NiO, ZnO, and Fe_3_O_4_ powders were ball milled at a speed of 600 rpm for 65 h at a ball/powder ratio of 10 : 1	–	326 at 10 mA cm^−2^	–	53.6	1	[41]

b.t.=before treatment; a.t.=after treatment.

**Table 2:** Summary of major oxygen‐deficient Co‐based TMO electrocatalysis for HER and HER/OER.^[a]^

Synthesis method	Material	Method	Overpotential (b.t.) [mV]	Overpotential (a.t.) [mV]	Tafel slope (b.t.) [mV dec^−1^]	Tafel slope (a.t.) [mV dec^−1^]	Ref.
hydrogen treatment	Co/Co_3_O_4_ core–shell nanosheets	pristine Co/Co_3_O_4_ core–shell nanosheets were annealed in H_2_ at 150, 200, and 300 °C for 3 h	302 at 20 mA cm^−2^	129 at 20 mA cm^−2^	49	44	[42]
thermal treatment	NiCo_2_O_4_ nanowire arrays	a precursor grown on carbon paper [prepared by hydrothermal treatment of a solution containing CoCl_2_ ⋅ 6H_2_O, NiCl_2_ ⋅ 6H_2_O, and CO(NH_2_)_2_] was annealed under air and Ar atmospheres at 350 °C for 2 h	294 at 10 mA cm^−2^	104 (Ar‐NiCo_2_O_4_) 226 (Air‐NiCo_2_O_4_) at 10 mA cm^−2^	_–_	112 (Ar‐NiCo_2_O_4_) 174 (Air‐NiCo_2_O_4_)	[43]
thermal treatment	Co_3_O_4_ nanoflowers	cobalt alkoxide precursor [obtained by hydrothermally reacting C_4_H_6_CoO_4_ ⋅ 4H_2_O, poly(*N*‐vinyl‐2‐pyrrolidone) and ethylene glycol solution (180 °C) for 3 h] was annealed in air (300 °C)	–	250 (HER) 297 (OER) at 10 mA cm^−2^	132.8 (HER) 108.4 (OER)	95.3 (HER) 79.1 (OER)	[44]
hydrogen treatment	NF/H‐CoMoO_4_ nanosheet arrays	pristine NF/CoMoO_4_ were annealed in H_2_/Ar at 400 °C for 1 h	223 (HER) 330 (OER) at 10 mA cm^−2^	42 (HER) 295 (OER) at 10 mA cm^−2^	117.2 (HER)	91 (HER)	[45]
hydrogen treatment	CoO/MoOx	pristine CoMoO_4_ was annealed in H_2_ at 500 °C for 2 h	353 (HER) at 10 mA cm^−2^ 370 (OER) at 20 mA cm^−2^	163 (HER) at 10 mA cm^−2^ 310 (OER) at 20 mA cm^−2^	61 (HER)	44 (HER)	[46]
plasma treatment	C−Co_3_O_4_	pristine Co_3_O_4_ nanosheets array was treated by CH_4_ plasma with different irradiation time (5–30 min), 300 W power 100 Pa	462 (HER) 340 (OER) at 10 mA cm^−2^	163 (HER) 250 (OER) at 10 mA cm^−2^	128 (HER) 68 (OER)	89 (HER) 54 (OER)	[47]
liquid exfoliation	CoFe LDH‐F	CoFe LDH was suspended in a flask containing DMF/ethanol; the suspension was vigorously stirred under Ar flow (24 h)	415 (HER) 345 (OER) at 10 mA cm^−2^	255 (HER) 300 (OER) at 10 mA cm^−2^	116 (HER) 47 (OER)	95 (HER) 40 (OER)	[48]
hydro‐thermal treatment	P‐doped Co_3_O_4_ nanosheet	pristine Co_3_O_4_/NF and NaH_2_PO_2_ with different weights (20, 60, 100, 140 mg) were heated at 300 °C for 0.5 h	165 (HER) at 10 mA cm^−2^ 315 (OER) at 20 mA cm^−2^	97 (HER) at 10 mA cm^−2^ 260 (OER) at 20 mA cm^−2^	129 (HER) 85 (OER)	86 (HER) 60 (OER)	[49]
plasma treatment	P‐Co_3_O_4_ nanosheet	NaH_2_PO_2_ and pristine Co_3_O_4_ were treated with Ar plasma at irradiation times 0–30 min at 200 W and 150 Pa	460 (HER) 340 (OER) at 10 mA cm^−2^	120 (HER) 280 (OER) at 10 mA cm^−2^	148 (HER) 70.8 (OER)	52 (HER) 51.6 (OER)	[50]
thermal treatment	CuCo_2_O_4_@carbon quantum dots	CuCo precursors [obtained via hydrothermal synthesis via reaction of carbon quantum dots, Cu(NO_3_)_2_ ⋅ 3H_2_O, Co(NO_3_)_2_ ⋅ 6H_2_O, and urea solution at 150 °C for 6 h] were annealed in Ar at 400 °C for 1 h	467 (OER) 420 (OER) at 10 mA cm^−2^	331 (HER) 290 (OER) at 10 mA cm^−2^	83 (HER) 90.3 (OER)	65 (HER) 64 (OER)	[51]

[a] All reactions performed in alkaline media (1 m KOH). b.t.=before treatment; a.t.=after treatment.

## 2 Mechanisms of Water Splitting

A detailed understanding of the HER and OER reaction mechanisms is critical to manufacturing electrocatalysts with high performance for efficient overall water splitting. The HER is a two‐electron transfer process and is facile under acidic conditions due to the availability of sufficient protons.^[8]^ Moreover, proton reduction is energetically more favorable in acidic compared to alkaline conditions.^[52]^ In alkaline HER, the protons (formed by the deprotonation of water molecules at the anode) combine with the excessive OH^−^ ions in alkaline solution, which hinders the HER reaction to proceed forward. Furthermore, HER reaction in alkaline media involves a kinetically slow initial water dissociation step before H^+^ formation, which introduces an additional energy barrier, thus resulting in approximately two orders of magnitude lower activity than in acidic media.^[1a]^ In general, the HER reaction mechanism in alkaline media consists of three reaction steps with two possible mechanisms;[^1c^, ^8^, ^53^] the first is the Volmer–Heyrovsky mechanism, and the second is the Volmer‐Tafel mechanism. The first step, so‐called the Volmer step [Eq. (1)], involves discharging proton from water on the electrocatalyst surface to form adsorbed hydrogen (H_ads_) intermediate. The second step is either electrochemical hydrogen adsorption of a second proton to form H_2_, known as the Heyrovsky pathway [Eq. (2)], or reaction of two adsorbed hydrogen atoms to form H_2_, known as the Tafel pathway [Eq. 3].[^1a^, ^54^]

(1) $$\mathrm{Volmer}\;\mathrm{reaction}:\;H{}_{2}O+e{}^{-}\to \mathrm{OH}{}^{-}+H{}_{\mathrm{ads}}$$

(2) $$\mathrm{Heyrovsky}\;\mathrm{reaction}\;H{}_{2}O+e{}^{-}+H{}_{\mathrm{ads}}\to \mathrm{OH}{}^{-}+H{}_{2}$$

(3) $$\mathrm{Tafel}\;\mathrm{reaction}:\;2\;H{}_{\mathrm{ads}}\to H{}_{2}$$

In acidic media, similar steps proceed to form molecular H_2_; however, during the Volmer reaction the protons are supplied from the hydronium cation (H_3_O^+^) instead of the water molecule, which combines with an electron to form adsorbed hydrogen (H_ads_) intermediate [Eq. (4)]. During the Heyrovsky reaction, the adsorbed hydrogen intermediate attracts an electron to produce a hydrogen molecule [Eq. (5)]. Similar to alkaline conditions, two adjacent absorbed hydrogen atoms chemically bond together to produce a hydrogen molecule via the Tafel reaction [Eq. 6].^[5]^

(4) $$\mathrm{Volmer}\;\mathrm{reaction}:\;H{}^{+}\left(\mathrm{aq}\right)+e{}^{-}\to H{}_{\mathrm{ads}}$$

(5) $$\mathrm{Heyrovsky}\;\mathrm{reaction}\;H{}^{+}\left(\mathrm{aq}\right)+e{}^{-}+H{}_{\mathrm{ads}}\to H{}_{2}$$

(6) $$\mathrm{Tafel}\;\mathrm{reaction}:\;2\;H{}_{\mathrm{ads}}\to H{}_{2}$$

The OER is a four‐proton‐coupled electron transfer process and is far more complex than the HER. In practice, the viability of the overall water splitting reaction is hindered by the high anodic OER overpotential.^[55]^ Understanding the mechanism of this complex reaction has been widely investigated in the literature and is of great importance to enhance OER performance.^[56]^ The kinetics of the OER in acidic and alkaline media vary depending on the material by which it is being catalyzed. In acidic media, OER is catalyzed more favorably by noble metal‐based catalysts than under alkaline conditions. In contrast, transition metal‐based materials catalyze the OER more easily in alkaline media than in acidic media.^[8]^

The first OER reaction step in alkaline media involves adsorption of the hydroxyl anion on the active site (M) and release of an electron to give M−OH [Eq. (7)]. Then, coupled proton and electron removals from M−OH takes place to form M−O [Eq. (8)]. Subsequently, oxygen formation follows two possible routes. In one route, M−O is converted to hydroperoxide M−OOH, and release of an electron after a hydroxyl anion is coupled. Then another proton‐coupled electron transfer process occurs to produce molecular O_2_ [Eqs. (9) and (10)]. In another route, a direct combination of two M−O species produces O_2_ [Eq. 11].^[57]^

(7) $$\mathrm{OH}{}^{-}\to M-\mathrm{OH}+e{}^{-}$$

(8) $$M-\mathrm{OH}+\mathrm{OH}{}^{-}\to M-O+H{}_{2}O+e{}^{-}$$

(9) $$M-O+\mathrm{OH}{}^{-}\to M-\mathrm{OOH}+e{}^{-}$$

(10) $$M-\mathrm{OOH}+\mathrm{OH}{}^{-}\to O{}_{2}+H{}_{2}O+e{}^{-}$$

(11) $$M-O+M-O\to O{}_{2}$$

It is worth noting that under acidic conditions, during the initial step, M−OH intermediate is formed by water oxidation rather than the oxidation of the hydroxide anion based on the following steps [Eqs. (12)–16].[^1c^, ^5^, ^12a^, ^13a^]

(12) $$H{}_{2}O\to M-\mathrm{OH}+e{}^{-}+H{}^{+}$$

(13) $$M-\mathrm{OH}\to M-O+e{}^{-}+H{}^{+}$$

(14) $$M-O+H{}_{2}O\to M-\mathrm{OOH}+e{}^{-}+H{}^{+}$$

(15) $$M-\mathrm{OOH}\to O{}_{2}+e{}^{-}+H{}^{+}$$

(16) $$M-O+M-O\to O{}_{2}$$

In general, most of the proposed OER mechanisms indicate the formation of M−O and M−OH intermediates regardless of the electrolyte conditions (alkaline or acidic). However, the process of molecular O_2_ formation can follow different routes that might or might not involve the formation of the M−OOH intermediate.[^1c^, ^54^] Every elementary step in the OER mechanism is associated with a kinetic energy barrier, which increases the overpotential required to drive the reaction. The elementary step with the slowest kinetics is regarded as the rate‐determining step (RDS) and affects the overall water‐splitting efficiency to a great extent.

## 3 Strategies for Preparing Oxygen‐Deficient Metal Oxides

Considering the performance improvements of oxygen‐deficient TMOs towards HER and OER, various studies have demonstrated several strategies to effectively create and tune the concentration of oxygen vacancies in TMOs without destroying the lattice or causing structural instabilities. This section will provide a thorough summary of synthesis strategies that have been developed to create oxygen vacancies in Co‐based TMO electrocatalysts including but not limited to hydrogen reduction, solution reduction, thermal annealing in an oxygen‐deficient environment, plasma treatment, and metallic and non‐metallic doping. The effect of synthesis method and process parameters on the surface morphology, crystallinity, and electrocatalytic HER and OER performances in terms of overpotential are highlighted.

### 3.1 Hydrogen reduction

**Figure 1:**
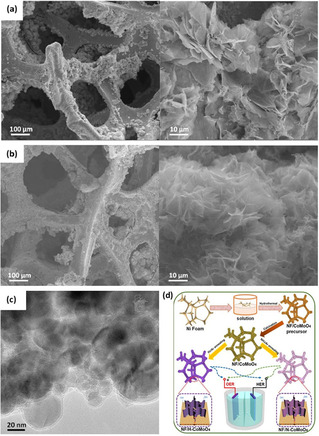
(a) SEM images of Co_3_O_4_ nanosheets (b, c) SEM and TEM images of Co/Co_3_O_4_ nanosheets, respectively, formed after heating of Co_3_O_4_ nanosheets in hydrogen at 200 °C for 3 h. Reprinted from Ref. [42] with permission from American Chemical Society, Copyright 2015. (d) Schematic illustration of the synthesis of 3D NF/H−CoMoO_4_ and NF/N−CoMoO_4_ electrodes for alkaline OER and HER catalysts. Reprinted from Ref. [45] with permission from Elsevier, Copyright 2020.

Hydrogen treatment at appropriate temperatures is commonly used to introduce oxygen defects in many metal oxides due to the high reducibility of hydrogen.[^12c^, ^58^] Mild reduction conditions can be implemented to introduce oxygen vacancies with an admissible degree of structural disorder.^[59]^ Yan et al.^[42]^ reported 3D Co/Co_3_O_4_ core‐shell nanosheets grown on Ni foam with abundant oxygen vacancies via reductive annealing treatment of nickel foam covered with Co(OH)_*x*_ in H_2_ atmosphere for 3 h. The concentration of oxygen vacancies was controlled by tuning the annealing temperature (150–300 °C), and the optimal temperature for the annealing process was found to be 200 °C. Post annealing, no distinct morphological changes were observed as the nanosheet maintained its self‐assembled flowerlike structures as shown in the SEM images displayed in Figure 1a, b. Moreover, annealing under a reductive H_2_ resulted in the formation of a thin amorphous layer (Figure 1c), thus the as‐synthesized Co/Co_3_O_4_ nanosheets consisted of a core‐shell structure with a crystalline metallic Co core and an amorphous Co_3_O_4_ and/or CoO shell. It was reported that the thin amorphous cobalt oxide layer was enriched with hydroxy groups, which contributed towards higher HER activity. The alkaline HER overpotential required to achieve 20 mA cm^−2^ current density was 129 mV using 3D Co/Co_3_O_4_ core‐shell nanosheets compared to Co_3_O_4_ nanosheets, which displayed an overpotential of 302 mV. As reported, the enhanced HER activity was also attributed to the synergetic effect of the metallic Co core and the amorphous Co_3_O_4_ and/or CoO shell, which facilitates surface activity and bulk conductivity.

A similar work reported by Chi et al.^[45]^ demonstrated a facile method to prepare oxygen‐deficient bifunctional CoMoO_4_ nanosheet arrays grown on 3D porous Ni foam (NF) denoted as NF/H‐CoMoO_4_ as shown in Figure 1d. Pristine NF/CoMoO_4_ nanosheet arrays were annealed in a reducing H_2_/Ar atmosphere at 400 °C. After annealing, NF/H‐CoMoO_4_ nanoplates preserved its structural integration of the pristine NF/CoMoO_4_. In contrast, the hydrogen treatment resulted in the exsolution of a secondary phase. The major phase in NF/H‐CoMoO_4_ was crystalline NF/CoMoO_4_ with a small amount of amorphous MoO_*x*_ phase. The introduction of surface oxygen vacancies enhanced the electrocatalytic activity of NF/CoMoO_4_ toward HER and OER performance. The overpotential to attain 10 mA cm^−2^ in alkaline solution was 42 and 295 mV for HER and OER, respectively. The latter values are substantially lower than the values obtained with NF/CoMoO4, which were 223 and 330 mV for HER and OER, respectively. Chi et al.^[45]^ reported that the formation of the amorphous component MoO_*x*_ contributed to the improved OER and HER activity of NF/H‐CoMoO_4_. Thus, besides the generation of oxygen defects, thermal annealing in a reductive environment in some cases can also lead to the formation of favorable secondary amorphous active phases, which can improve the activity of metal oxides.

Other reductive gaseous atmospheres such as NH_3_ were also explored in some works to create oxygen vacancies under high processing temperatures.[^45^, ^60^] Although hydrogen treatment is by far the most efficient strategy to generate oxygen defects in metal oxides among reductive gases, it involves highly explosive hydrogen gas, which is not favorable as it may pose potential safety hazards that limit its industrial scalability.

### 3.2 Solution reduction

Oxygen vacancies in Co metal oxides can also be created via wet‐chemical/solution reduction using reducing agents including, but not limited to, sodium borohydride (NaBH_4_), hydrazine (N_2_H_4_), and titanium chloride (TiCl_3_). Solution reduction can take place either at room temperature or by going through mild thermal processing.[^7^, ^12a^, ^12b^, ^61^]

**Figure 2:**
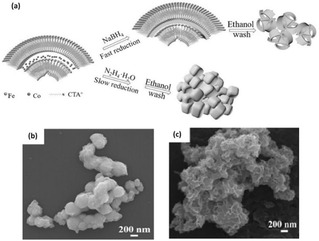
(a) Schematic diagram of the preparation of Fe_1_Co_1_−ONS and Fe_1_Co_1_−ONP. SEM images of (b) Fe_1_Co_1_−ONP and (c) Fe_1_Co_1_−ONS. Reproduced from Ref. [29] with permission from WILEY‐VCH Verlag GmbH & Co. KGaA, Weinheim, Copyright 2017.

The effect of reduction rate on the intensity of oxygen vacancies in iron‐cobalt oxide nanosheets (Fe_*x*_Co_*y*_‐ONS) using two different reducing agents (NaBH_4_ and N_2_H_4_) was studied by Zhuang et al. (Figure 2a).^[29]^ They reported that N_2_H_4_ showed lower and slower reducibility than NaBH_4_ and resulted in the formation of the Fe_1_Co_1_ oxide nanoparticle (Fe_1_Co_1_−ONP) (Figure 2b). The fast reduction by NaBH_4_, provides homogeneous dispersion of the metal ions to avoid phase separation, thus maintaining the nanosheets structure (Figure 2c). This has resulted in inducing more oxygen vacancies in Fe_1_Co_1_‐ONS than Fe_1_Co_1_−ONP. The Fe_1_Co_1_−ONS structure promotes facile mass diffusion/transport of OH^−^ ions and provides higher active surface area (more exposed active sites) for OER compared to Fe_*x*_Co_*y*_−ONP. Moreover, oxygen vacancies lowered the energy barrier of OH^−^ anions adsorption during the electrocatalytic reaction, which has a preferential effect on OER activity. An alkaline OER overpotential of 308 mV was required to reach a current density of 10 mA cm^−2^ for the reduced Fe_1_Co_1_−ONS, compared to 400 mV for the reduced nanoparticles counterpart.

Synthesis of oxygen vacancy‐rich Co_2_AlO_4_ nanosheets via solvothermal approach was successfully achieved by Wang et al.,^[30]^ by treating pristine Co_2_AlO_4_ nanosheets in a solution of sodium hydroxide (NaOH) and ethylene glycol at 140 °C for 12 h. The reduced Co_2_AlO_4_ exhibited a higher surface area (54.1 m^2^ g^−1^) compared to pristine Co_2_AlO_4_ (31.6 m^2^ g^−1^). implying the availability of more active sites for OER. Additionally, the ratio of Co^+2^/Co^+3^ on the surface of reduced Co_2_AlO_4_ (1.78) was significantly higher than that of pristine Co_2_AlO_4_ (0.84) and hence contained higher oxygen vacancies. Co^+2^ ions are accountable for the formation of cobalt oxyhydroxide (CoOOH), which is an ideal active intermediate for OER activity. Moreover, the generated oxygen vacancies on Co_2_AlO_4_ nanosheets were found to enhance the adsorption of water onto nearby Co^+3^ sites, which promotes OER performance. Thus, the overpotential of reduced Co_2_AlO_4_ in alkaline solution at 10 mA cm^−2^ current density was 280 mV, which is remarkably lower than its pristine counterpart Co_2_AlO_4_ (360 mV).

The solution reduction method is highly scalable, comparatively safer, and more affordable than high‐temperature annealing under reductive hydrogen as it enables the generation of oxygen‐deficient metal oxides at ambient or mild conditions and, therefore, does not involve the risk of explosion.

### 3.3 Thermal annealing in an oxygen‐deficient environment

**Figure 3:**
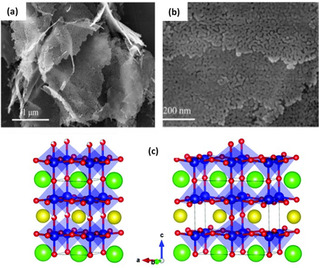
(a) SEM image of Co_3_O_4_ nanoflowers. (b) TEM image of Co_3_O_4_ nanoflowers. Reproduced from Ref. [44] with permission from Elsevier, Copyright 2020. (c) Schematic crystal structures of PrBaCo_2_O_5.75_ (left) and PrBaCo_2_O_5.5_.(right) Color codes: Pr=orange, Ba=green, Co=blue, O=red. Reproduced from Ref. [26] with permission from The Royal Society of Chemistry, Copyright 2019.

Thermal annealing of metal oxides at elevated temperatures in an inert atmosphere (e. g., air, Ar, N_2_, or their mixture), oxygen‐deficient environment, or vacuum leads to the loss of oxygen atoms, thereby creating oxygen defects.[^12b^, ^59^, ^62^] Du et al.^[44]^ prepared Co_3_O_4_ nanoflowers with a single‐layer porous structure and with enriched oxygen vacancies by thermal annealing of cobalt alkoxide precursor, which was obtained via hydrothermal reaction of C_4_H_6_CoO_4_ ⋅ 4H_2_O, poly(*N*‐vinyl‐2‐pyrrolidone), and ethylene glycol solution at 180 °C for 3 h in air at 300 °C. After annealing, Co_3_O_4_ maintained its nanoflower‐like (Figure 3a) and porous structure (Figure 3b), thus providing more surface area of the electrocatalytic active sites. The as‐synthesized Co_3_O_4_ nanoflowers showed remarkable OER and HER performance in alkaline media where an overpotential of 297 and 250 mV was required to reach 10 mA cm^−2^ current density, respectively.

The effect of annealing temperature on the concentration of surface oxygen vacancy in cobalt oxide perovskite and its role in OER was reported by Miao et al.^[26]^ Two oxygen‐deficient cobalt oxide perovskites with different oxygen vacancy concentrations (*δ*) of 0.25 (PrBaCo_2_O_5.75_) and 0.5 (PrBaCo_2_O_5.5_) were prepared by annealing pristine PrBaCo_2_O_6‐*δ*_ in pure N_2_ atmosphere for 30 min at 250 and 600 °C, respectively. Increasing the annealing temperature facilitated the formation of oxygen vacancies. However, the concentration of vacancies was found to have a detrimental effect on OER kinetics. PrBaCo_2_O_5.75_ displayed higher OER activity in alkaline media with a low overpotential of 360 mV at 10 mA cm^−2^ compared to PrBaCo_2_O_5.5_ (420 mV). Structural analysis revealed that at higher annealing temperatures (more oxygen defects), the oxygen vacancies tend to orderly align in PrO_1‐*δ*_ (Figure 3c). This ordered structure lowers the cobalt oxidation states and causes a spin‐state transition from high‐spin to low‐spin states for cobalt ions, which both significantly hinder the OER kinetics.

Although thermal annealing in an inert and oxygen‐deficient environment requires elevated annealing temperatures, it is a fairly straightforward process and requires a simple experimental setup. Therefore, this method provides a facile and affordable route to process oxygen‐defective metal oxide electrocatalysts of high throughput.

### 3.4 Plasma treatment

Plasma treatment is a form of surface etching and has been considered to be a fast and effective method toward the preparation of oxygen‐deficient metal oxides.^[28]^ During plasma activation, the bombardment of high energetic particles/ions (Ar^+^, N_2_^+^, H_2_O^+^) with the metal oxide surface engraves the oxide to expose more surface active sites and breaks covalent bonds on the surface. This causes lattice surface atoms to produce oxygen vacancies.^[63]^ Furthermore, a certain level of heteroatom doping occurs, which as well further enhances the generation of vacancies. Besides physical etching, plasma treatment using reactive gases (e. g., H_2_) causes chemical etching that activates reactions of the gas radicals with surface atoms to induce vacancies.^[64]^

Xu et al.^[33]^ followed a one‐step facile method to introduce oxygen vacancies on Co_3_O_4_ nanosheets using N_2_ plasma treatment for different irradiation times at 300 W power and 40 Pa pressure. Morphological analysis revealed that pristine Co_3_O_4_ nanosheets exhibited a continuous and compact structure. However, after plasma activation for 60 s at 300 W, the surface of the Co_3_O_4_ nanosheets became discontinuous, loose, and porous, resulting in a larger surface area and a greater number of active sites. No notable transformation of the Co_3_O_4_ bulk phase was observed after the plasma treatment, indicating that oxygen vacancies were only generated on the surface of Co_3_O_4_. Moreover, the plasma engraved Co_3_O_4_ nanosheets showed surface enrichment of N species, thus simultaneous N doping and etching of Co_3_O_4_ nanosheets to produce N‐doped nanoporous Co_3_O_4_ (N−Co_3_O_4_) nanosheets occur during plasma treatment. This further adjusts the electronic states for Co_3_O_4_ nanosheets, which improves the electrocatalytic conductivity thus contributes to higher OER activity. N−Co_3_O_4_ nanosheets required an OER potential of 1.54 V in alkaline media to attain a current density of 10 mA cm^−2^. On the other hand, the pristine nanosheets required 1.79 V, indicating the remarkable OER activity of the plasma‐engraved nanosheets.

**Figure 4:**
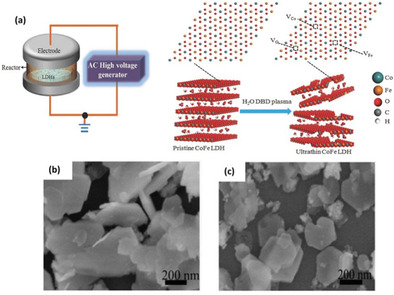
(a) Schematic illustration of the water‐plasma‐enabled exfoliation of CoFe LDH nanosheets. The DBD plasma reactor is designed with the plate‐to‐plate electrode at 50 V powered by the AC high voltage generator. SEM images of (b) pristine CoFe LDHs and (c) water‐plasma exfoliated CoFe LDH nanosheets. Reproduced with permission from Ref. [34] with permission from WILEY‐VCH Verlag GmbH & Co. KGaA, Weinheim, Copyright 2017.

Besides dry plasma treatment, Liu et al.^[34]^ used H_2_O‐plasma treatment in a dielectric barrier discharge (DBD) plasma reactor to obtain defected ultrathin CoFe layered double hydroxide (LDH) nanosheets from pristine CoFe(LDH) nanosheets as shown in Figure 4a. During plasma treatment, the electrostatic forces between metal layers and interlayer cations are broken to exfoliate CoFe(LDH) nanosheets and metal hydroxide layers with induced oxygen vacancies. Besides inducing oxygen vacancies, efficient exfoliation of the pristine CoFe(LDH) nanosheets stacking structure was achieved as shown in Figure 4b, c, which facilitated exposure of the electrocatalytic active sites and enhanced OER activity. Moreover, other vacancies were generated including Co and Fe vacancies, which also contribute to higher electrocatalytic performance. The plasma‐treated ultrathin CoFe(LDH) nanosheets required a low overpotential of only 290 mV at 10 mA cm^−2^ compared to pristine CoFe(LDH) nanosheets (332 mV).

The creation of oxygen vacancies via plasma activation has proven to be highly efficient as the treatment can be accomplished in a short time and the concentration of oxygen vacancies can be fine‐tuned by varying the plasma power, pressure, gas flow, and/or irradiation time. However, the scalability of this technique is limited due to the high cost of plasma generation equipment and power supply.

### 3.5 Heteroatom doping

Apart from the in situ ion doping, element doping is also an effective route to enhance the intrinsic electronic properties of metal oxides by introducing heteroatoms into crystal lattice to create an unbalanced charge atmosphere, leading to the formation of oxygen vacancies to maintain thermodynamic stability. Various metallic and non‐metallic dopants (e. g., P, Sr, B, Fe, N) have proved to be efficient in modulating the electronic properties of metal oxides.[^58^, ^62^]

**Figure 5:**
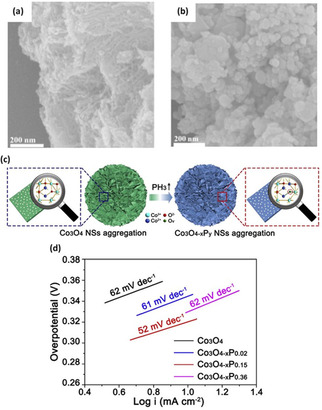
(a, b) SEM images of B‐doped CoO−Ov and CoO. Reproduced from Ref. [24] with permission from WILEY‐VCH Verlag GmbH & Co. KGaA, Weinheim, Copyright 2018. (c) Schematic plot of controllably introducing O_*v*_ into mesoporous Co_3_O_4_ NSs aggregation by P‐doping strategy. (d) Electrocatalytic activities of Co_3_O_4_, Co_3_O_4_‐_*x*_P_0.02_, Co_3_O_4‐*x*_P_0.15_, and Co_3_O_4‐*x*_P_0.36_. Reproduced from Ref. [35] with permission from Elsevier, Copyright 2018.

Oxygen‐deficient B‐doped CoO−O_*v*_ nanowires were synthesized by Zhang et al.^[24]^ through thermally annealing a mixture of Co(Ac)_2_ and NH_4_HB_4_O_7_ at 500 °C for 2 h in Ar. For comparison, pure CoO was prepared by hydrothermal treatment of a solution containing cobalt(II) chloride hexahydrate and ammonium acetate. Post annealing, the morphology of pristine CoO transformed from an uneven aggregate‐like structure to a wire‐like structure (Figure 5a, b). Consequently, deficient B‐doped CoO−O_*v*_ nanowires exhibited a higher specific surface area compared to pristine CoO. Moreover, the presence of low‐valence Co cations was detected in B‐doped CoO−O_*v*_ as compared to that in CoO and Co(OH)_2_. The latter is typically an indication of the presence of oxygen vacancies. Electrochemical performance analysis under alkaline conditions revealed that the overpotential to reach 10 mA cm^−2^ current density was 280 mV compared to 400 mV for undoped pristine CoO. Zhang et al. reported that the enhancement in electrocatalytic activity is attributed to the incorporation of oxygen vacancies via B‐doping, which lowered the kinetic energy barrier required to break the chemical bonding between Co and O, thus achieving highly active OER performance.

Xu et al.^[35]^ fabricated phosphorous (P)‐doped mesoporous Co_3_O_4_ nanosheets with tunable oxygen vacancies via hydrothermal synthesis route. Co_3_O_4_ nanosheets and NaH_2_PO_2_⋅H_2_O with different weight ratios (2.0, 15.0, 30) were annealed in a low‐pressure Ar atmosphere at 250 °C for 1 h (Figure 5c). During annealing, thermal decomposition of NaH_2_PO_2_⋅H_2_O forms PH_3_ reductive gas, creating P atom doping and oxygen vacancies in Co_3_O_4_ nanosheets. Crystallographic structure analysis indicated that upon increasing the P‐doping content (Co_3_O_4‐*x*_P_0.15_), a CoO cubic phase forms and by further adding P‐dopant (Co_3_O_4‐*x*_P_0.3_), the phase transformation from cubic CoO phase to orthorhombic CoO occurs. As reported, the latter transformation was due to the reducibility effect of PH_3_ gas. On the other hand, doping preserved the surface morphology of pristine Co_3_O_4_ nanosheets. The optimized sample (Co_3_O_4−*x*_P_0.15_) illustrated high activity towards OER in alkaline media and delivered a current density of 20 mA cm^−2^ within a small overpotential of 338 mV, which is lower than that for the undoped Co_3_O_4_ (395 mV) (Figure 5d).

### 3.6 Other methods

Besides the aforementioned techniques, other means to introduce oxygen vacancies in Co‐based electrocatalysts have been reported. Kang‐Wen et al.^[37]^ employed laser fragmentation to fabricate FeCo_2_O_4_ nanoparticles with abundant oxygen vacancies. The oxygen‐defected FeCo_2_O_4_ nanoparticles (R−FeCo_2_O_4_) were synthesized by irradiating FeCo_2_O_4_ microspheres (M−FeCo_2_O_4_) dispersed in deionized water at room temperature for 30 min using nanosecond pulsed Nd:YAG laser source. Laser ablation in liquid environments could provide high temperature and rapid heating/cooling to facilitate the generation of oxygen vacancies. The as‐prepared R−FeCo_2_O_4_ showed a remarkable activity towards OER as it delivered an overpotential of 276 mV at the current density of 10 mA cm^−2^; this value is lower than the value of pristine FeCo_2_O_4_ microspheres (297 mV). This method is a physical process and does not require the use of chemical precursors or surfactants, which enables the formation of clean defected surfaces with abundant oxygen vacancies. Moreover, the process can be performed within short periods due to the high temperature available at the moment of laser irritation followed by fast cooling.

**Figure 6:**
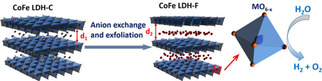
Schematic representation of material structures. After being vigorously stirred in the DMF/ethanol solution, the high‐affinity CO_3_^2−^ interlayers of the CoFe LDHs have been dissolved and exchanged to HCOO^–^ ions. The consequent exfoliated process exposes more oxygen vacancies as active sites and improves the electronic conductivity. Note: d_1_ and d_2_ are the interlayer distances, d_2_>d_1._ Reproduced from Ref. [48] with permission from American Chemical Society, Copyright 2016.

Liquid‐phase exfoliation technique was reported by Liu et al.^[48]^ to fabricate oxygen‐deficient CoFe LDH−F nanosheets from bulk CoFe LDH‐C. Exfoliated CoFe LDH nanosheets were prepared by vigorously stirring bulk CoFe LDH‐C in a mixture containing dimethylformamide/ethanol under Ar flow for 24 h. The hydrolysis of DMF‐ethanol results in the formation of formates for continuous decarbonation and delamination of the LDH catalyst. Upon vigorous stirring of the liquid suspension, the solvent penetrates the CoFe‐LDH structure resulting in breaking the binding forces between the integrated hydrogen network. Consequently, leading to a loosely stacked exfoliated structure (larger interlayer spacing) as shown in Figure 6. The atoms on the surface of the electrocatalyst are displaced from the lattice upon exfoliation, thus inducing oxygen vacancies. For the bulk CoFe LDH‐C, alkaline overpotential values of 266 and 300 mV were required to achieve 10 mA cm^−2^ HER and OER current density, respectively. On the other hand, oxygen‐deficient CoFe LDH‐C displayed a much lower overpotential value of 255 and 300 mV for HER and OER, respectively. Although liquid exfoliation is a facile technique to generate oxygen‐deficient nanosheets, in some cases adsorption of the solvent molecules on the synthesized product may occur, which blocks the active sites and deters the electrocatalytic performance.

A rather unexplored oxygen vacancy induction method has also been recently reported by Zhang et al.,^[14b]^ whereby a precisely engineered Co_3_O_4_ layer with a controllable degree of oxygen vacancies was synthesized on a Nickel foam substrate through a solid‐phase melting approach. It was shown that the oxygen vacancy content atop Co_3_O_4_ can be easily tuned by adjusting reaction temperature. A medium oxygen vacancy content (32 %) has resulted in the highest mass activity (−2.12 A g^−1^) at an overpotential of 200 mV, in comparison to the samples with low (22 %) and high (45 %) oxygen vacancy contents. It only required ultralow alkaline HER overpotential of 203 mV to achieve an apparent current density of −100 mA cm^−2^. In another study, Zhang et al.^[65]^ developed oxygen‐deficient and facetted hollow pompon‐like Co_3_O_4_ nanostructures through a self‐assembled encapsulation‐calcination method. A mere 370 mV overpotential was required for OER to reach 100 mA cm^−2^ current density in alkaline media, much lower than those of Co_3_O_4_ nanowires.

## 4 Characterization Techniques for Oxygen Vacancies

Anionic point defects, in particular oxygen vacancies, are produced by the loss of an oxygen atom from the metal oxide lattice. A structural phase transformation may occur depending on the material type and the degree of lattice parameter change upon relaxation. Direct probing of oxygen vacancies has been challenging; however, several techniques can be employed to indirectly investigate changes induced by oxygen vacancies. Generally, these include crystal structure changes, electronic differences in metallic and oxygen species within the crystalline lattice near oxygen vacancy sites, and magnetic property changes as a result of surface charge neutrality effects. This section groups and discusses characterization techniques based on electron microscopy, photonics analysis, and analytical methods.

### 4.1 Electron microscopy

**Figure 7:**
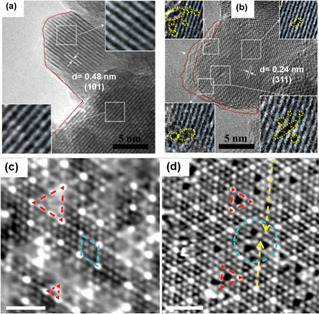
(a, b) HRTEM image of CMO and CMO‐4. The insets of (a) and (b) show magnified view of perfect and distorted lattices in (a) CMO and (b) CMO‐4, respectively. The red dotted lines indicate smooth and rough edges of particles in CMO and CMO‐4. The blue lines [insets of (a) and (b)] highlight crystallographic planes, while the yellow dotted circles/eclipses indicate intra/inter‐crystalline sites and surface oxygen vacancies. Reproduced from Ref. [66] with permission from Elsevier, Copyright 2017. (c) STM image for the formation of oxygen vacancies in CeO_2_(111)/Pt (111) of the imperfect 3 : 4 lattice match in monolayered CeO_2_(111)/Pt (111), V=−0.27 V, I=0.20 nA, scale bar=2 nm. Mismatched and 3 : 4 matched domains are marked by red‐dashed triangles and blue‐dashed rhombus, respectively. (d) STM image of the O vacancies (red dashed triangles) formed in the mismatched areas (blue dashed circles), V=−0.11 V, I=1.2 nA, scale bar=2 nm. Reproduced from Ref. [67] with permission from American Chemical Society, Copyright 2020.

Emergent structural distortions can be probed by contrasts in the atomic arrangements taken by high‐resolution transmission electron microscopy (HRTEM) and scanning transmission electron microscopy (STEM). Sadighi et al.^[66]^ detected oxygen vacancies by magnifying the HRTEM images as shown by the insets of white squares in Figure 7a, b where the atomic planes are highlighted by blue lines. These insets clearly indicate perfect crystalline planes of Co−Mn−O nanocubes and reticular Co_*x*_Mnx_3‐*x*_O_4_ (CMO and CMO‐4, respectively) while several vacancies, specified by yellow dotted circles/eclipses, are obvious among CMO‐4 crystalline planes.

Scanning tunneling electron microscopy (STM) has also been successfully used to determine the presence of lattice oxygen vacancies on materials surfaces. Direct surface imaging at an acutely small length scale by scanning a sharp metal wire tip over a surface has been reported to pull oxygen vacancies from the bulk to the surface and detect them.[^67^, ^68^] Formation of oxygen vacancies from CeO_2_ grown on Pt (111) generated imperfect 3 : 4 monolayer CeO_2_(111)/Pt(111) structures, which were recently identified using STM by Rong et al.^[67]^ (Figure 7c, d). Although this technique has not yet been explicitly utilized for cobalt‐based materials, the approach applied to oxygen vacancies from CeO_2_ grown on Pt (111) inspires extending its application to cobalt‐based electrocatalysts.

### 4.2 Photonics analysis

**Figure 8:**
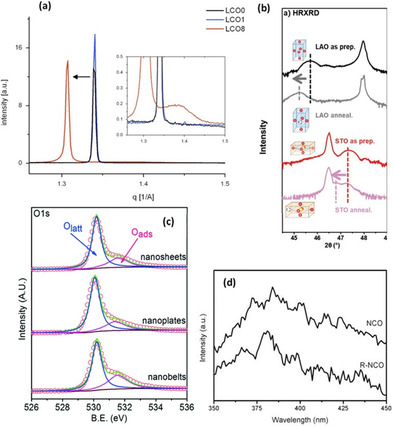
(a) Effect of chemical doping leads to significant change in (003) peaks. Inset: close up of (003) peak, X‐ray data (SWAXS). Reproduced from Ref. [69] with permission from Elsevier, Copyright 2014. (b) HRXRD 2θ−ω scans of LSC films in their as‐prepared states and after vacuum annealing. The arrow showed the shift of the film peaks after vacuum annealing, indicating the chemical expansion introduced by oxygen vacancy formation. Reproduced from Ref. [73] with permission from WILEY‐VCH Verlag GmbH & Co. KGaA, Weinheim, Copyright 2019. (c) XPS spectra of O 1s of all as‐prepared Co_3_O_4_ catalysts. Reproduced from Ref. [74] with permission from The Royal Society of Chemistry, Copyright 2016. (d) Room‐temperature PL spectra of pristine and reduced NiCo_2_O_4_ (NCO) at 300 nm excitation wavelengths. Reproduced from Ref. [75] with permission from American Chemical Society, Copyright 2018.

Crystallinity structural defects can be induced upon formation of oxygen vacancies due to natural relaxations of the lattice upon a vacancy formation. A decreased crystallite dimensionality of nanomaterials can result which can be characterized by comparing the pristine and reduced crystallinity of a material. Basch et al.^[69]^ showed subtle peak shifts and width broadening in Rietveld refinement patterns of synchrotron wide‐angle X‐ray scattering (WAXS) in Figure 8a. Such studies proved useful for the successful exfoliation of commercially available Li_*x*_CoO_2_ (named as LCO0), whereby water‐treated and HCl‐treated samples of this material were prepared, namely LCO1 and LCO8, respectively. The phase transition indicated by structural distortions in the LCO8 sample at Li_*x*_=0.34 from peak broadening in Figure 8a allowed for analogous comparison with graphite intercalates, and the potential employment of similar materials for two‐dimensional heterostructuring. Similarly, Chattot et al.^[70]^ found that upon potential surface atomic debuckling of PtNi nanostructure due to dissolution of non‐noble metal (dealloying), surface or near‐surface vacancies are introduced. Lattice expansion/contraction and phase transformation can be quickly probed using X‐ray diffraction (XRD), and this has been previously reported in several studies that induced a structural volume change.[^62^, ^71^] Induction of oxygen vacancies in several perovskite structures has been probed using high‐resolution (HR) XRD.[^59^, ^72^] Liu et al.^[73]^ utilized HR XRD for detecting out‐of‐plane changes in lattice parameters for La_0.7_Sr_0.3_CoO_3‐*δ*_/LaAlO_3_ (LAO) and La_0.7_Sr_0.3_CoO_3‐*δ*_/SrTiO_3_ (STO) thin films after vacuum annealing as shown in Figure 8b. Peaks shift to lower angles upon annealing (and consequent introduction of oxygen vacancies) infers an increase in out‐of‐plane lattice parameter and unit cell volume. Due to the large number of electrons becoming available upon oxygen vacancy formation, the atomic underbonding orbitals in the transition metal result in an increased out‐of‐plane lattice parameter(s) and consequently cell volume.^[59]^

X‐ray photoelectron spectroscopy (XPS) has been a prominent tool in determining the surface electronic and chemical states of materials by effectively measuring the contrast in outermost layer electrons and their atomic cores’ binding energies. Oxygen vacancy peaks of adsorbed oxygen species are typically found in oxygen‐deficient materials upon the deconvolution of the O 1s peak. Figure 8c shows two fitted peaks for the O 1s spectra of oxygen‐deficient Co_3_O_4_ nanostructures. Typically, deconvolution yields a main peak corresponding to lattice oxygen and a secondary peak for adsorbed oxygen at binding energies around 531.4 eV. The latter is composed of O_2_^−^ or O^−^, and both are strong electrophilic species that could attack parts of water molecules captaining the highest electron densities. In their work, Jiang and co‐workers^[74]^ indicated that oxygen deficient Co_3_O_4_ sample showed the highest activity compared to non‐treated Co_3_O_4_ samples, and correlated its activity to the highest degree of stable oxygen vacancies. Furthermore, Bera et al.^[72b]^ confirmed the existence of Co^2+^/Co^3+^ using XPS on oxygen‐deficient Co_3_O_4_ nanosheets upon solution‐based NaBH_4_ reduction of pristine Co_3_O_4_ nanosheets. Thus, reduction in surface cation valence states can be measured by XPS in order to cope with surface charge electroneutrality conditions for more thermodynamic surface‐stable reduced materials.

Photoluminescence spectroscopy (PL) is another well‐spread method that is utilized for characterization techniques for studying electronic changes of materials and is utilized to determine the degree of oxygen deficiency in metal oxides. The presence of oxygen vacancies generates new electronic states near the Fermi level and generally modulates the bulk electronics of a material Also, anionic vacancies typically modify band structures and result in alternating paths for excited electron generation and recombination, which occurs from light‐directed electron excitation during PL readings. Peak position and intensity of the PL spectra can easily identify changes that occur upon such electronic modulations from oxygen vacancies. Figure 8d shows that notable changes are evident in the PL spectra upon reduction of pristine (NCO) and reduced (R‐NCO) spinel NiCo_2_O_4_.^[75]^ Oxygen deficient R‐NCO demonstrates a stronger PL emission peak at 380 nm than pristine NCO when using the excitation wavelength of 300 nm. This behavior is related to the recombination of holes with two electron trapped oxygen vacancies.

**Figure 9:**
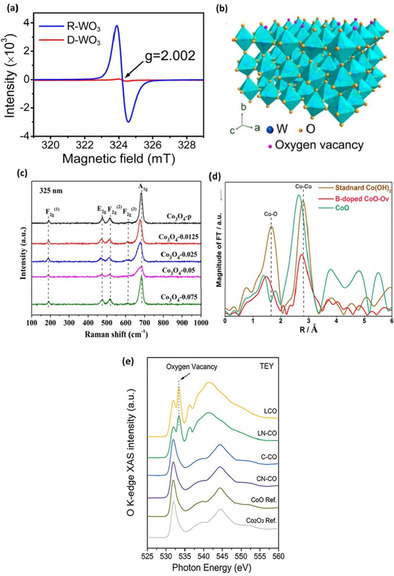
(a) Room‐temperature ESR spectra and (b) schematic illustrating the locations of oxygen vacancies in WO_3_ lattice. Reproduced from Ref. [76] with permission from American Chemical Society, Copyright 2016. (c) Raman spectra at the excitation wavelength of 325 nm of Co_3_O_4_‐p and OV‐Co_3_O_4_. Reproduced from Ref. [79] with permission from American Chemical Society, Copyright 2019. (d) FT spectra at the Co K‐edge for B‐doped CoO−Ov, CoO, and standard Co(OH)_2_. Reproduced from Ref. [24] with permission from WILEY‐VCH Verlag GmbH&Co. KGaA, Weinheim, Copyright 2018. (e) Surface‐sensitive TEY sXAS scans at the O K‐edge of L−CO, LN−CO, C−CO, CN−CO, and reference CoO, Co_2_O_3_. The emerging new peak at approximately 533.5 eV on the spectra of L−CO and LN−CO refers to oxygen vacancy. Reproduced from Ref. [81] with permission from WILEY‐VCH Verlag GmbH & Co. KGaA, Weinheim, Copyright 2019.

Electron paramagnetic spectroscopy (EPR) provides finger printing information of bulk and surface unpaired electrons, which is a strong indicator of oxygen vacancies. For instance, Zhang et al.^[76]^ showed oxygen deficiency in reduced WO_3_ (R‐WO3) via EPR that depicted a typical g parameter of 2.002 for R‐WO_3_ (Figure 9a, b). Presence of oxygen vacancies traps unpaired electrons to absorb the recorded resonance. Several researchers have utilized EPR to characterize the presence of anionic point defects in a myriad of materials, and all have resulted in a g parameter of 2.00.^[77]^ However, EPR limitation emerges from its inability to distinguish between different types of defects (i. e., structural pits, cationic and anionic vacancies).

Acquiring information pertaining to molecular vibrations and rotations via Raman spectroscopy is another tool that has been widely adopted for identifying oxygen‐deficient materials. Varying chemical bonds upon induction of oxygen vacancies results in different vibrational modes. This change can produce lattice molecular vibrational level changes, which give rise to Raman shifts. Defect structures in metal oxides can thus impact vibrational modes and generate Raman shifts or new peaks entirely. Park and co‐workers^[78]^ engineered transition metal‐doped Co_3_O_4_ for electrochemical OER and found through XPS, TEM, and Raman spectroscopy that the doped samples include surface oxygen vacancies. Wang et al.^[79]^ studied NO reduction performance effects upon introducing oxygen vacancies in porous Co_3_O_4_ via NaBH_4_ solution reduction. Figure 9c shows a representation of the Raman intensity peaks at 189, 465, 510, 602, and 667 cm^−1^ corresponding to symmetric stretching of the Co−O bond in the octahedral sites of the cubic lattice. The A1 g symmetry of the oxygen‐deficient samples, correlating to the highest intensity peak at 667 cm^−1^, reveal a negative shift that is indicative of oxygen vacancies induction. The doped samples convey a clear redshift (an increase in wavelength), which may originate from expansion and disordering of crystal lattice by doping of the TM cations. This simple and inexpensive technique has been applied to several oxygen‐deficient materials.^[80]^

X‐ray absorption spectroscopy (XAS) is widely adopted method for studying the local geometric configurational changes and electronic structures of solids. Recently, X‐ray absorption near edge structure (XANES), a sub‐method of XAS, has been gaining traction as a feasible and accurate method in determining valence states of the adsorbing atom. Extended X‐ray absorption fine structure (EXAFS) is another reported sub‐category of XAS, which essentially probes the local chemical and electronic microenvironment on a material's surface. Liu and co‐workers^[24]^ reported efficient OER activity upon introduction of oxygen vacancies in CoO by the incorporation of boron doping. Fourier transform (FT) of EXAFS *k* ^3^ [χ(*k)*] at the Co K‐edge is displayed in Figure 9d. Co K‐edge oscillation curves indicate that the B‐doped CoO‐Ov demonstrated much weaker EXAFS oscillations compared with CoO and standard Co(OH)_2_ samples. This suggests that the local structure of the B‐doped CoO−Ov electrocatalyst was markedly more disordered due to structural and electronic modulations from oxygen vacancies. Moreover, oxygen vacancies can be investigated by measuring the total electron yield intensity (TEY) from soft XAS (sXAS). Figure 9e presents the O−K edge spectra of several cobalt oxides. Du and co‐workers^[81]^ reported that sXAS spectra of cobalt oxides synthesized by laser ablation (LCO and LN‐CO) show a peak at 533.4 eV, which is assigned to oxygen vacancies. This peak is not present for cobalt oxides synthesized under a wet‐chemical scheme. This indicates that oxygen vacancies are more effectively formed under the former laser ablation procedure.

### 4.3 Other techniques

Iodometric titration (IT) was utilized by Wang et al.^[82]^ to determine the stoichiometry of oxygen in SrCo_0.9_Ta_0.1_O_3‐*δ*_. Primarily, the material is powdered and dissolved in HCl solution with excess potassium iodide (KI). Na_2_S_2_O_3_ solution was titrated into the above solution in order to determine the average oxidation state of Co ions. Sr and Ta were assumed to have fixed oxidation states of +2 and +5, respectively. Governed by charge electroneutrality conditions to be satisfied, the oxygen stoichiometry (lattice oxygen) and the degree of oxygen vacancies can be quantified.

Positron annihilation spectroscopy (PAS), also called positron annihilation lifetime spectroscopy (PALS), has also been reported for use in identifying oxygen vacancies in metal oxides. This characterization technique rapidly picked up in its reported utilization shortly after the discovery of oxygen deficient and disordered “black” TiO_2_. Briefly, when positrons are emitted into a solid sample, they interact with the present electrons and annihilate rapidly releasing gamma rays in a given recorded time (typically in the order of ≈1 ns). In the case of defects (i. e., voids or oxygen vacancies), the time it takes for annihilation is retarded, and as such the presence of these defects can be recorded. This method has been successfully used in identifying oxygen vacancies in hydrogenated TiO_2_ and cobalt oxides (i. e., CoO).^[83]^

All the aforementioned discussed techniques show a varying degree of accuracy and reliability in determining the presence of oxygen vacancies. However, based on our literature review of which characterization methods seem to be most accurate and reliable with respect to corresponding oxygen‐deficient TMO works, a pattern emerges. XPS, STM, XAS (i. e., EXAFS and XANES), and HRTEM seem to be the most reliable, followed by XRD, EPR, and IT. A major drawback to the excessive utilization of STM in more TMO characterization exists in the lack of high conductivity and ultra‐clean surfaces typically exhibited in TMOs, even those with surface oxygen deficiencies. XPS analyses innately limits characterization of oxygen vacancies in the first few surface layers. XRD studies of oxygen vacancies are only appropriate when dealing with highly crystalline materials. However, as will be discussed later in this work, several amorphous secondary active surface phases can exhibit oxygen vacancies and are primary sources of higher electrochemical activity in their respective materials. Thus, factoring for the fact that every technique exhibits some form of limitation, it has been accepted as a best practice to use multiple characterization techniques on the same material to undoubtedly prove the presence of oxygen vacancies.

## 5 Role of Oxygen Vacancies on the Electrocatalytic Activity

The generation of surface oxygen vacancies can effectively modulate the surface morphology, electronic structure, and thus electrocatalytic properties of TMOs. Presence of these vacancies can markedly modify the interaction processes between the catalyst and reactants. Experimental and computational studies over the past decade have shed light on mechanistically explaining how oxygen vacancies can ameliorate high overpotentials in HER and OER of electrocatalytic metal oxides. Predominantly, three features were found to contribute to enhancements in performance, namely: changes in transport properties, the favorable formation of secondary active surface phases, and modulation of the bulk electronic structure. All of the aforementioned effects have a complementary impact on adsorption and desorption of reaction intermediates, potentially lowering rate‐determining thermodynamic and kinetic barriers. Generally, in water splitting the OER is limiting due to higher overpotentials as a result of it entailing 4‐e^−^ step, compared to 2‐e^−^ steps for HER. Furthermore, an overwhelming majority of OER catalysts, including the benchmark IrO_2_ and RuO_2_, show poor stability in acidic media; TMOs tend to be utilized and investigated more for the alkaline OER process. Acidic conditions promote a lower overpotential for HER; however, due to metal oxide stability issues, not many are investigated for HER. Notwithstanding, this section will offer a comprehensive summary of experimental and computational explanations put forth from literature to better construe how oxygen vacancies promote TMOs electrocatalytic water splitting.

### 5.1 Changes to charge transport

Anion defects such as oxygen vacancies were directly related to enhance the electronic conductivity in TMOs. This design aspect is important since having a limiting charge transport would render an intrinsically active surface a poor electrocatalyst, consequentially decreasing OER performance.^[61]^ It has been acknowledged that electronic conductivity in TMOs is quite poor, substantially limiting their potential electrocatalytic activities.^[84]^ Ling et al.^[85]^ identified through experimental and theoretical techniques that OER activity of oxygen‐vacant rock salt CoO synthesized as single crystal was significantly improved. It was determined that one of the main intrinsic factors that improved due to localized oxygen vacancies on {111}‐O facets of single crystal CoO was in fact the carrier concentration compared with its typical polycrystalline CoO counterpart.

**Figure 10:**
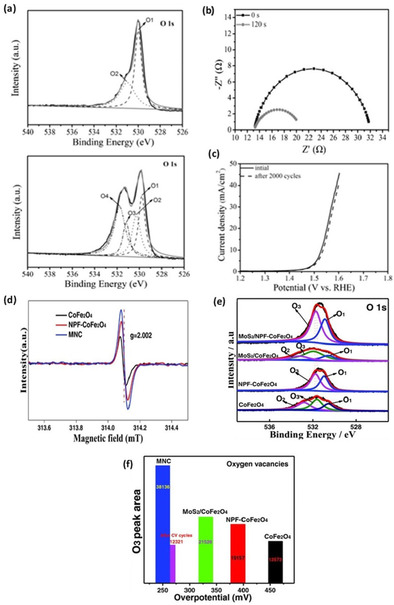
(a) Fitted O 1s XPS spectra, (b) OER Nyquist plot, and (c) LSV stability polarization curve of OER on pristine and plasma‐engraved reduced Co_3_O_4_. Reproduced from Ref. [86] with permission from WILEY‐VCH Verlag GmbH & Co. KGaA, Weinheim, Copyright 2016. (d) EPR spectra with a g factor of 2.002, (e) fitted O 1s XPS spectra, and comparison between oxygen vacancy (O3) XPS peak area, (f) OER overpotentials [mV] of spinel CoFe_2_O_4_, N, P, and F tri‐doped (NPF), CoFe_2_O_4_ (NPF−CoFe_2_O_4_), and MoS_2_/NPF−CoFe_2_O_4_ (MNC). Reproduced from Ref. [88] with permission from WILEY‐VCH Verlag GmbH & Co. KGaA, Weinheim, Copyright 2018.

The high availability of different coordination surface oxygen in spinel type TMOs promoted them as ideal candidates for enhancing OER performance upon introduction of oxygen vacancies. Wang et al.^[16b]^ reported an effective reduced nanosheets of Co_3_O_4_ spinel type OER electrocatalyst fabricated by a plasma‐engraving procedure. This method did not only improve the intrinsic activity of Co_3_O_4_ by modifying the electronic states, but also by increasing the surface area through formulation of a porous surface, which exposed more active sites. Presence of oxygen vacancies was confirmed through a comparative analysis of the pristine and reduced spinel‐type TMO by employing XRD and XPS measurements. O 1s spectra from XPS analysis of plasma‐engraved reduced Co_3_O_4_ nanosheets in Figure 10a show the presence of a satellite peak corresponding to the binding energy of oxygen vacancies in the lattice surface.^[86]^ A large amount of defect states was found to be present in the bandgap of the modified Co_3_O_4_. From a rather simplistic view, the ease of transference of two electrons existing at an oxygen vacancy defect state to the conduction band (CB) can be directly related to increased conductivity. Nyquist plots of OER on pristine and reduced spinel Co_3_O_4_ in Figure 10b convey a smaller semicircle for the oxygen‐vacant sample, indicating lower charge transfer resistance (CTR) and consequently higher conductivity. Figure 10c depicts a rather favorable OER overpotential of 300 mV was adequate for the plasma‐engraved Co_3_O_4_ nanosheets to obtain current density of 10 mA cm^−2^ and it showed very good stability after 2000 linear sweep voltammetry (LSV) cycles. This was 240 mV lower than pristine nanosheets due to the introduction of oxygen vacancies. As with many materials reported in literature, the enhancement in intrinsic catalytic performance of a material tends to be a combination of more than one fundamental aspect. In the case of plasma‐engraved reduced Co_3_O_4_, a combination of higher conductivity and exposed surface area resulted in substantial decrease in the required OER overpotential.

Through calculations of density of states (DOS) from density functional theory (DFT) approaches, Xiao et al.^[50]^ showed that the presence of oxygen vacancies in Co_3_O_4_ reduces the bandgap from approximately 1.5 to 0.8 eV. The OER thermodynamic overpotential for pristine Co_3_O_4_ was experimentally measured to be 470 mV at 10 mA cm^−2^. However, only 420 mV was required upon introduction of a single oxygen vacancy in their multi‐slab simulation. Experimentally determined HER performance was also shown to improve for the oxygen‐deficient spinel oxide, which was attributed to an enhanced conductivity.

Formation of oxygen vacancy in perovskite‐type materials such as NiCo_2_O_4_, Sr_2_Fe_1.3_Ni_0.2_Mo_0.5_O_6‐*δ*_, and Ca_0.9_Yb_0.1_MnO_3_ were also shown to enhance electronic conductivity and consequently improve OER activity.[^25^, ^72b^, ^87^] Furthermore, Lyu et al.^[40]^ measured electronic conductivity of CaMnO_3‐*δ*_ and observed its increase from 3.33×10^−^3 Sm^−1^ to 2.19×10^−2^ Sm^−1^ upon increasing the concentration of oxygen vacancies. Incorporation of a spinel ferrite, namely CoFe_2_O_4_, and electroactive 2D MoS_2_ has been successfully achieved by Sun et al.^[88]^ to yield an amorphous and massively oxygen‐deficient MoS_2_/NPF‐CoFe_2_O_4_ (MNC). The material emerges as one of the most active spinel‐type‐based OER electrocatalysts, with a reported overpotential (at 10 mA cm^−2^) of 250 mV and small Tafel slope of 41 mV dec^−1^. Figure 10d depicts the EPR signal for MNC showing a g factor of 2.002 corresponding to oxygen vacancies on TiO_2_ and ZrO_2_ catalysts.[^88^, ^89^] As discussed in section 4.2, an EPR signal with a g factor of approximately 2 typically correlates to oxygen vacancies. Essentially, EPR readings at high g factors is an indication of electrons trapped on oxygen vacancy sites, thereby implying the presence of oxygen vacancies for MNC. XPS measurements were employed to quantify oxygen vacancies from the area under the peak at a binding energy of 531.6 eV.^[90]^ The measured oxygen vacancy (O3) peak areas and their corresponding overpotentials are shown in Figure 10e, f. In a similar work by Zhao et al.,^[91]^ 2D ultrathin MnO_2_ nanosheets were Co‐doped, resulting in an interestingly oxygen deficient

Co−MnO_2_|O_v_ nanostructure with an OER overpotential (at 10 mA cm^−2^) of 279 mV. The improvement of OER activity of the Co‐doped oxygen‐deficient material was attributed to the decrease in charge transfer resistance (essentially increase in conductivity) based on the Nyquist plots and DOS calculations. Projected DOS calculations show that Co−MnO_2_|O_v_ has more states occupying energy levels near the Fermi level, which suggests a higher conductivity for the oxygen‐vacant material.

The synergetic work between DFT techniques and laboratory experimental has thus proven invaluable in proposing novel materials with stellar performance for the field of electrochemistry including, but not limited to, electrocatalysis, and pseudo‐capacitance. The effective utilization of DFT as a screening tool for unique materials guided by proven activity descriptors that target tunable bulk properties, such as conductivity, will lead to the rapid fabrication and testing of rationally designed electrocatalysts. Furthermore, the seemingly untrodden path of integrating machine learning (ML) techniques based on quantum mechanics (QM) calculations and experimental data will substantially curb the presently large computational times. Investigating the performance of material groups for applications using ML approaches will also result in huge databases. The integration of data mining methods to the aforementioned databases may sequel the discovery of more accurate activity descriptors and ultra‐high performing materials. Coupling this knowledge with ever‐improving synthesis techniques that are both controllable and benign can usher a revolution in surface micro‐engineering of catalysts.

### 5.2 Formation of secondary active surface phases

Presence of surface oxygen vacancies on TMOs can generate secondary active surface phases, which can have preferential effects on the electrocatalytic activity of the resultant material. Secondary active phases have been reported through the utilization of thermal annealing, plasma treatment, and solution‐based redox methods.[^33^, ^92^] The latter two have been given attention lately due to the controllable formation of surface metal oxyhydroxides (i. e., MOOH, where M represents a TM) known to act as bifunctional catalytic surfaces for overall water splitting. Wang and co‐workers reported a solution based hydrothermal oxidation method producing Zn‐substituted CoOOH (Zn_0.2_Co_0.8_OOH) using Zn and Co metal−organic frameworks (MOFs) as precursors.^[93]^ The Zn_0.2_Co_0.8_OOH sample, which shows presence of surface oxygen vacancies based on XPS analysis, also corresponded to the lowest theoretical overpotential of 270 mV for the OER among other solids. Two computational OER mechanisms were investigated in order to determine the reaction coordinate of the respective materials. The conventional adsorbate evolution mechanism (AEM) limits the theoretical OER overpotential to 370 mV due to the integration of multiple adsorbed intermediates exhibiting highly correlated adsorption strengths.^[94]^ The lattice‐oxygen oxidation mechanism (LOM) was recently identified based on perovskite oxides as a viable mechanism that involves direct O−O coupling and bypasses AEM limitations.^[95]^ Thus, in the work of Wang and co‐workers with Zn_*x*_Co_1‐*x*_OOH, both mechanisms that were examined using DFT approaches to formulate a reaction coordinate diagram. Experimental OER overpotentials at current density of 10 mA cm^−2^ in alkaline media were consistent with the theoretical predicted values of 385 mV for CoOOH and 241 mV for Zn_0.2_Co_0.8_OOH. Interestingly, increasing the Zn/Co ratios from 0.3 to 0.5 increased the overpotentials from 285 to 351 mV. This indicates that for representative TM oxyhydroxides, an optimum surface oxygen vacancy ratio exists that ameliorates a preferential secondary surface phase.

**Figure 11:**
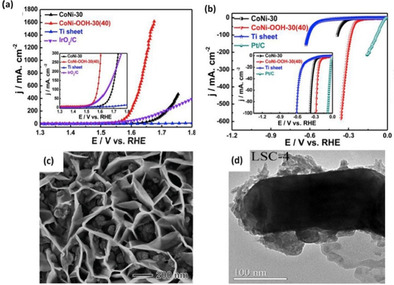
(a, b) Alkaline (1.0 m KOH) a) OER and b) HER LSV polarization curves of bimetallic CoNi, CoNi oxyhydroxide, and benchmark IrO_2_/C and Pt/C electrodes, respectively, recorded at a scan rate of 5 mVs^−1^. Reproduced from Ref. [96] with permission from Elsevier, Copyright 2019. (c) HRSEM image of oxygen vacant FeCoOOH nanosheets grown on nickel foam (NF). Reproduced from Ref. [97] with permission from WILEY‐VCH Verlag GmbH & Co. KGaA, Weinheim, Copyright 2018. (d) TEM image of La_0.8_Sr_0.2_CoO_3‐*δ*_−4 (LSC‐4) showing grey shaded regions corresponding to the presence of a hydrous oxide secondary active phase with oxygen vacancies. Reproduced from Ref. [98] with permission from Elsevier, Copyright 2017.

Yu et al.^[96]^ found that the electrodeposition of a nickel‐cobalt alloyed ridge‐like structure followed by alkaline electrooxidation method resulted in a very stable bifunctional CoNi oxyhydroxide (CoNi−OOH) material with apparent surface oxygen vacancies. HER and OER overpotentials at 10 mA cm^−2^ registered at −210 and 279 mV, respectively, and low Tafel slopes of 67 and 62 mV dec^−1^ (see Figure 11a, b). Nguyen et al.^[97]^ immersed a reduced porous nickel foam (NF) in an iron‐cobalt nitrate aqueous mixture and hydrothermally treated it to obtain a FeCoOOH oxyhydroxide nanosheets (see Figure 11c). O 1s spectra from XPS also reveal the presence of the representative oxygen vacancy peak at approximately 532.3 eV for both TM alloy oxyhydroxides.

Amorphous phase formation of perovskite materials also becomes a secondary active surface phase demonstrating good OER activity. Lam et al.^[98]^ treated La_0.8_Sr_0.2_CoO_3−*δ*_ (LSC) with 30 % aqueous H_2_O_2_ and found that OER was strongly enhanced. The performance improvement was attributed to the formation of an amorphous active secondary surface (hydrous oxide) phase with oxygen defects upon LSC surface treatment by the peroxide. OER activity of LSC‐*X* (*X* denoting number of consecutive peroxide treatment cycles) increases with *X*. The current density at 1.70 V vs. RHE was measured to be 6.03, 16.30, 22.31, 27.72, and 39.94 mA cm^−2^ for *X* values of 0, 1, 2, 3, and 4, respectively. HRTEM imaging of LSC‐4 shows the light shaded hydrous oxide surface in Figure 11d. Thus, although oxygen vacancies present in secondary active phases show an improvement in electrochemical performance, the effect is usually a combination of several factors including surface and bulk energetic modifications. The utilization of in situ characterization for monitoring the formation of active secondary phases may shed light on more controllable synthesis techniques that can be implemented on existing promising catalysts.

### 5.3 Modulation of the bulk electronic structure

**Figure 12:**
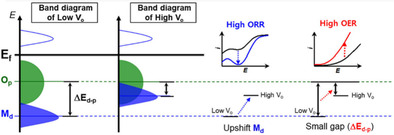
Schematic rigid band diagrams of the late TMO. Reproduced from Ref. [101] with permission from with permission from American Chemical Society, Copyright 2020.

Substantial efforts have been poured into identifying what are known as activity descriptors based on an electrocatalyst's bulk electronic properties. Correlation between such activity descriptors and the established RDS inspired two main attributes, essentially M 3d and O 2p energy distances (Δ*E*_d‐p_) and the e_g_ filling theory. In brief, a smaller energy gap between the metal 3d and oxygen 2p band center of the TMO leads to reduced charge transfer barriers (enhanced conductivity) and stronger covalent character between the metal and oxygen, which improves OER.^[99]^ Oxygen vacancies with smaller energy penalties exist when a higher O 2p band center relative to the Fermi level is present, corresponding to a reduced OER overpotential in perovskites. Grimaud et al.^[100]^ showed that substituting trivalent cations such as La^3+^ on the A‐site of perovskites with divalent Sr^2+^ resulted in a reduction between M 3d and O 2p band centers, whereby favoring oxygen vacancy creation and OER performance improvement. The formation of O−O bonds is much more energetically favorable on SrCoO_3_ compared with LaCoO_3_, explaining the more sanctioned OER performance for SrCoO_3_. Kim and co‐workers^[101]^ primarily focused on investigating the relationship between oxygen vacancies, Δ*E*_d‐p_, and apparent overpotential for Sm_0.5_Sr_0.5_CoO_3‐*δ*_ (SSC). The authors were motivated by the fact that previous studies solely focused on the effect of *δ* on catalytic activity without much consideration of the electronic structure of cobalt in SSC. Early TMs (i. e., V, Mn, and Cr) show higher metal d‐band center (M_d_) compared to the oxygen p‐band center (O_p_), while late TMs (i. e., Ni, Co, and Cu) show lower M_d_ compared to O_p_.^[102]^ Thus, OER performance can be increased by increasing M_d_ which in effect decreases the Δ*E*_d‐p_. This relationship is displayed in Figure 12. Similarly, Wu et al.^[103]^ found that partial substitution of Al by Fe into CoAl_2_O_4_ activates the original material through surface reconstruction into Co oxyhydroxides with an apparent uplifting of the oxygen 2p levels, which facilitate oxygen deficiencies and enhance the catalyst activity. This resulted in a considerable reduction of the OER overpotential by approximately 70 mV for CoFe_0.25_Al_1.75_O_4_ relative to CoAl_2_O_4_, at 10 μA1 cm^−2^.

**Figure 13:**
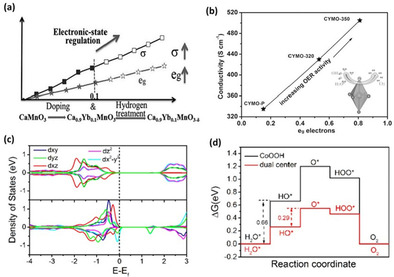
(a) Electronic state regulation of ABO_3_ perovskite catalyst optimization of number of electrons at Mn e_g_ orbital and corresponding conductivity (σ) by light doping and hydrogen treatment and (b) OER activity increase with respect to σ and e_g_ electron filling (inset: Jahn‐Teller distortion promoting oxygen vacancy formation, resulting in optimal Mn e_g_ filling and better conductivity). Reproduced from Ref. [87] with permission from WILEY‐VCH Verlag GmbH&Co. KGaA, Weinheim, Copyright 2015. (c) PDOS of cobalt in CoOOH (top) and oxygen‐vacant Zn‐CoOOH (bottom) and (d) OER free energy diagram of single‐ and dual‐center models corresponding to CoOOH and Zn−CoOOH. Reproduced from Ref. [104] with permission from Elsevier, Copyright 2018.

Further e_g_ filling (i. e., d*z* ^2^, d*x* ^2^−*y* ^2^) is another OER activity descriptor for the electronic structure of TMOs, and e_g_ filling approaching 1 generally shows optimum OER performance. Guo et al.^[87]^ found that introduction of oxygen vacancies in CaMnO_3_ perovskite‐type TMOs through direct hydrogen atmosphere calcination leads to an initial increase in conductivity, followed by a decrease after a certain threshold is reached that renders the structure too unstable. The e_g_ filling states continued to monotonically increase with this method. A similar behavior was also observed when doping with ytterbium up to *x*=0.2 (Yb_0.2_Ca_0.8_MnO_3_). Interestingly, as can be seen from Figure 13a, initial doping followed by hydrogen treatment, both the e_g_ filling and conductivity can be tuned to optimize OER activity. In their work, the alkaline OER electrocatalytic activity of optimum Yb_0.1_Ca_0.9_MnO_3_ was 100 times higher than that of the pristine undoped and untreated CaMnO_3_ sample. This was attributed to optimal Mn e_g_ filling state as high as 0.81 and improved conductivity as well. The corresponding results are presented in Figure 13b.

Wang et al.^[104]^ reported that the incorporation of the lower valence‐state Zn on Co oxyhydroxide (Zn−CoOOH) leads to lower formation energies for oxygen vacancies and induced Jahn‐Teller distortions. This effect improved the lift degeneracy of t_2g_ (i. e. d*xy*, d*xz*, d*yz*) and e_g_ orbitals, leading to a partially occupied d*z* ^2^ orbital, which resulted in more thermodynamically favorable water adsorption step, as can be seen from Orbital DOS in Figure 13c. Faster kinetics are also achieved under the same effect due to hydrogen bond formation, triggering dual activity center on the electrocatalyst surface, which facilitated proton transfer and dissociation. The free‐energy landscape of single and dual‐center models is presented in Figure 13d. XPS measurements before and after OER activity confirmed the stability of the oxygen vacancies, and the structural conformation at large. The overpotentials required to attain a current density of 10 mA cm^−2^ for pristine CoOOH and Zn−CoOOH were approximately 270 and 310 mV, respectively.

**Figure 14:**
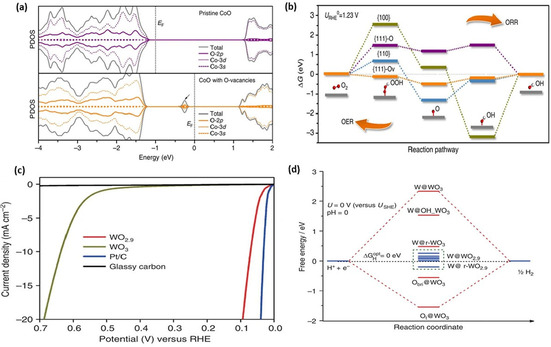
(a) PDOS of pristine CoO and oxygen‐vacant CoO (black arrow points towards newly created electronic states near the Fermi level in CoO with oxygen vacancies, responsible for adsorption of intermediates on the oxygen vacancies sites) and (b) calculated ORR/OER free‐energy coordinate energy diagram at the equilibrium potential (1.23 V vs. RHE) on different CoO facets. Reproduced from Ref. [85] with permission from Springer Nature, Copyright 2016. (c) LSV polarization curves for oxygen vacant WO_2.9_, WO_3_, and benchmark Pt/C for the HER and (d) calculated free‐energy diagram of HER at the equilibrium potential for a series of active sites on (001) WO_3_ and (010) WO_2.9_. Reproduced from Ref. [105] with permission from Springer Nature, Copyright 2015.

Investigating the effect that electronic structure has on {111}‐Oxygen vacant SC CoO was performed by Ling et al.^[85]^ through the employment of DFT techniques. As indicated by the PDOS in Figure 14a, the introduction of oxygen vacancies generates new electronic states via Co‐3d, Co‐3s, and O‐2p hybridization in the CoO bandgap. The newly created electronic states are closer to the Fermi level than existing states prior to oxygen vacancies, and this has been generally accepted to be a predominant cause in enhanced electronic conductivity and stronger binding of OER intermediates. Figure 14b depicts the strengthening of intermediate free binding energies (Δ*G*) between {111}‐O terminated SC CoO and its oxygen deficient form (i. e., {111}‐O_v_}). This decrease in the Δ*G* between OER intermediates steps is a direct result of stronger and more stable binding with the newly electronically modulated oxygen vacant surface, resulting in much more favorable thermodynamic overpotentials.

As can be inferred from the aforementioned catalysts, most cobalt‐based electrocatalysts have higher activities for OER than HER. Although several cobalt‐based materials have been found to be bifunctional for both the OER and HER, the innate characteristics of cobalt generally make it un‐preferential towards HER. Nonetheless, motivated by the vast interest in oxygen deficient cobalt based oxides, we were enthralled by finding increasing interest and work in oxygen‐deficient tungsten‐based oxides specifically for HER. For instance, Li et al.^[105]^ carried out structural analyses and theoretical calculations to compare WO_3_ and its reduced analogue WO_2.9_. Their results indicated formation of new electronic states near the Fermi level for WO_2.9_. Experimentally, it was found that the recorded acidic HER overpotential at 10 mA cm^−2^ registered 637 and 70 mV for WO_3_ and WO_2.9_, respectively, as shown in Figure 14c. This considerable HER activity improvement was investigated through the employment of DFT whereby the generally accepted HER descriptor (i. e., Gibbs free energy of hydrogen binding, Δ*G*_H_) was used. Figure 14d shows that active catalytic sites for the reduced WO_2.9_ model exhibit absolute Δ*G*_H_ value much closer to the ideal thermodynamic value of zero. It is important to appreciate that a Δ*G*_H_≫0 indicates poor binding, limiting the initial adsorption of hydrogen in the Volmer step of the HER reaction mechanism. Contrarily, a Δ*G*_H_≪0 indicates stronger binding, limiting the final reassociation and dissociation Tafel step that releases H_2_ gas from the active surface. It can be inferred from Figure 14d that both metal (i. e., W) and oxygen (i. e., top and bridge) sites for the oxygen‐vacant WO_2.9_ have all been modulated to more favorable hydrogen adsorption energies, leading to the lower observed overpotential for WO_2.9_.

## 6 Conclusion and Outlook

With a rapid increase in employment of surface engineering defects of transition metal oxides (TMOs), and in particular oxygen vacancies, fundamental understanding of the different micro‐effects at play is crucial for further advancements of material design and synthesis in the field of electrocatalysis. We were drawn towards cobalt‐based oxides with oxygen deficiencies due to their low overpotentials for the oxygen evolution reaction (OER) that have been recently reported by many researchers. Kinetic limitations in near neutral pH electrocatalytic water splitting can be curbed by highly modulating the intrinsic activity of stable and earth abundant cobalt oxides. This would allow for more environmentally benign electrolysis and stable operation of electrolyzers. This Review primarily focused on high perception in design, synthesis/modulation, and characterization of oxygen‐deficient cobalt‐based electrocatalysts. Also, the role of oxygen vacancy and suggested mechanisms for improving electrocatalytic activities were discussed. Our literature search showed that cobalt oxides were identified as primary targets for surface modifications to create oxygen vacancies toward high OER electrocatalytic activity. This work provided an in‐depth discussion of how oxygen defects can lead to more favorable surface charge redistributions and create local charge aggregation around the vacancy site. Changes in transport properties, formation of secondary active surface phases, and modulation of the bulk electronic structure and their respective effects on electrocatalytic activity were examined. A myriad of available TMO group types such as spinel, rock salt, layered double hydroxide, trivalent oxide, perovskite, and others are available for further studies.

It is essential to highlight that the major contribution that oxygen vacancies have towards performance improvement is to ultimately weaken, or strengthen, the binding energy of reaction intermediates to the electrocatalyst's surface. Thus, a rational employment of oxygen vacancy engineering should be undertaken. We accentuate that the synthesis of electrocatalysts should be preceded by a rational design based on fundamental understanding of how and what electronic, transport, and phase modulations are desired. Computationally aided theoretical calculations like density functional theory (DFT) have been widely adopted and accepted to initially screen and interpret enhancements in activity obtained upon introduction of oxygen vacancies. In this work, we point out the importance of DFT in identifying and appropriating intrinsic (i. e., new near‐Fermi level electronic states) and reaction‐based [i. e., Δ*G*_H*_ for hydrogen evolution reaction (HER), Δ*G*_(O, OH, OOH)*_ for OER] activity descriptors.

It is worth noting that further investigation in other types of vacancy defects, both metallic and non‐metallic, may lead to development of highly active OER and HER materials. Defect engineering of carbonaceous materials such as graphene and graphdiyne (a new 2D carbon allotrope analogues to graphene) and utilizing them as supports for effective electrocatalysts can result in interesting phenomena in various applications, including water splitting. The careful examination of stability effects on electrocatalytic materials with defects is essential in the feasible implementation of these materials to commercial‐grade technologies. The hand‐to‐hand exploitation of machine learning, quantum mechanics, and experimental techniques to screen, characterize and study potential methods for stability enhancement is expected to revolutionize the scale of potential materials for water splitting applications. It is recommended that further studies using the environmentally benign, timely, and controllable utilization of plasma treatment strategies (particularly for cobalt and tungsten oxides) are to be undertaken. Development of in situ techniques to characterize formation, location, content, and stability of oxygen vacancies and other point defects will prove very fruitful for the subsequent modeling and modulation of electrocatalysts.

## Conflict of interest

The authors declare no conflict of interest.

## Biographical Information

*Ahmed Badreldin obtained his M.Sc. in Chemical Engineering from Texas A&M University at Qatar in 2019. He is currently working on his PhD degree in Chemical Engineering at Texas A&M University. He has also been involved in the development of photocatalytic materials for water treatment and splitting, and on modelling systems of solid polymeric electrolytes using Mixed Anion Techniques for Lithium Ion Battery Development. Currently, Ahmed is planning on completing his PhD degree with primary focus on developing a highly functional seawater electrolyzer with novel durable and highly active electrocatalysts for industrially feasible implementation through experimental, DFT, and ML approaches*.

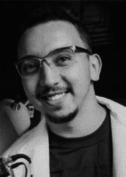

## Biographical Information

*Aya E. Abusrafa obtained her M.Sc. in Chemical Engineering from Texas A&M University at Qatar in 2019. She is currently pursuing her PhD degree in Chemical Engineering from Texas A&M University. Her current research focus is on experimental electrocatalyst fabrication for overall water splitting. She was previously involved in computational fluid dynamic modeling of Fischer–Tropsch reactors using a novel Micro fibrous bed under nonconventional reaction media and surface modification of polymeric materials using plasma treatment*.

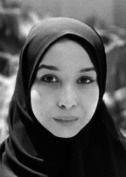

## Biographical Information

*Prof. Ahmed Abdel‐Wahab is a Professor of Chemical Engineering at Texas A&M University at Qatar and a professor of Civil Engineering at Texas A&M University. His research primarily focuses on chemical, electrochemical, and photochemical processes associated with water and wastewater treatment, carbon dioxide reduction, and water splitting. This research has led to publication of more than 110 peer‐reviewed articles in leading research journals, 9 book chapters, and more than 80 refereed conference publications/presentations*.